# Vestibular migraine. Clinical and diagnostic challenges, and emerging therapeutic approaches

**DOI:** 10.1097/WCO.0000000000001447

**Published:** 2025-12-02

**Authors:** Maria Dolores Villar-Martinez, Ahmed Abdalla, Peter J. Goadsby

**Affiliations:** a NIHR King's Clinical Research Facility, SLaM Biomedical Research Centre and Wolfson Sensory Pain and Regeneration, Institute of Psychiatry, Psychology and Neuroscience, King's College, London, UK; b King Abdullah University of Science and Technology, Thuwal, Saud Arabia; c Neurology and Psychiatry Department, Minia University, Minia, Egypt

**Keywords:** comorbidities, phenotyping, treatment, vertigo, vestibular migraine

## Abstract

**Purpose of review:**

Vestibular migraine (VM) is a prevalent yet underdiagnosed cause of vestibular symptoms, which overlaps with other vestibular and migraine-related conditions. This review focuses on detailed clinical phenomenology, alongside comorbidities, and the appraisal of emerging therapies.

**Recent findings:**

Recent work shows that migraine-associated features such as allodynia, photophobia, and movement sensitivity sharpen clinical discrimination. Premonitory and cognitive symptoms, including brain fog and executive slowing, are increasingly recognized. Chronobiological factors such as menstrual cycle and menopause modulate susceptibility. Oculomotor assessment and neuroimaging point to disturbed integration across vestibular, sensorimotor, and visual networks rather than focal lesions. Comorbid persistent postural-perceptual dizziness, dysautonomia, and autoimmune tendencies complicate diagnosis and management. Early trials support calcitonin gene-related peptide (CGRP) monoclonal antibodies and onabotulinumtoxin-A, with lifestyle interventions, and nutraceuticals commonly being used, although clinical trial designs and endpoints remain heterogeneous.

**Summary:**

VM reminds us that bedside examination remains the anchor: a detailed history, eye-movement examination, and context refine diagnosis. Objective markers and interdisciplinary strategies assist rather than replace clinical judgement. Further studies should integrate multimodal assessment and phenotype-guided treatment stratification.

## INTRODUCTION

Vestibular migraine (VM) is a complex neuro-otological disorder characterized by vertigo and other altered perceptions of space and motion in patients with a history of migraine [1–3], often accompanied by nonspecific vestibular and sensory symptoms that may overlap with other vestibular and migraine-related conditions [4^▪^].

This review focuses on recent clinical and translational research on the role of clinical phenomenology and oculomotor signs, then consider central vestibular processing, autonomic dysfunction, neuroimaging, and comorbidities in shaping the VM phenotypes, and cross-specialty treatment approaches. 

**Box 1:**
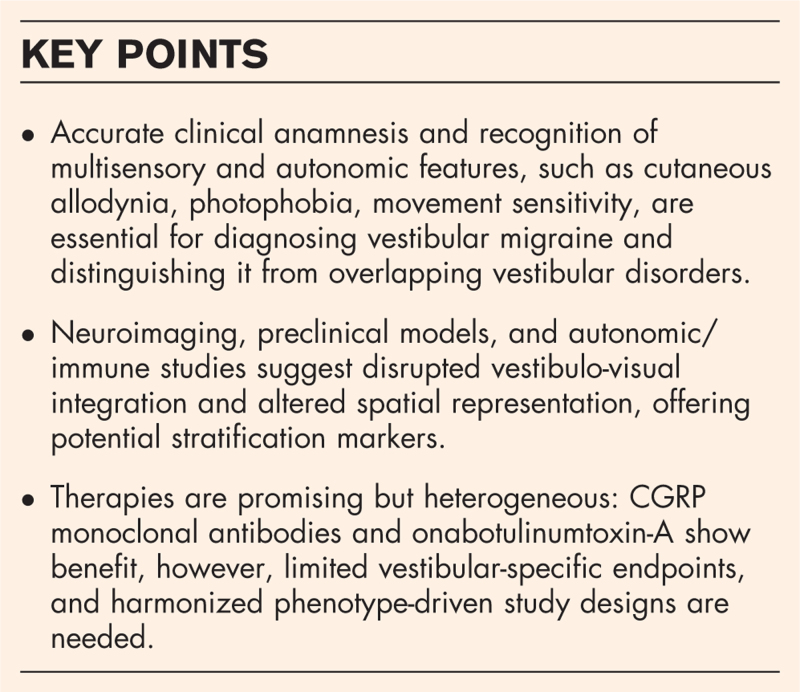
no caption available

## CLINICAL PHENOTYPING, VARIATION OVER TIME, AND EXAMINATION

Features not totally captured by existing criteria, such as movement sensitivity, and contextual clues like a positive family history of migraine, can help to clarify a VM diagnosis [5]. Context, time-course, and triggers, rather than any single test, often carry diagnostic weight. The presence of cutaneous allodynia may imply more severe attacks [6^▪^], and has been linked to higher odds of VM diagnosis [7]. Patients with photophobia may experience more visually-induced vertigo [8], and sensitivity to light or visual stimuli could worsen a potential conflict between visual and vestibular input [9]. A recent study in 400 VM patients did not discriminate between VM subgroups classified according to phonophobia, photophobia and/or visual aura [10], underscoring that absence of abnormality on standard vestibular tests does not exclude VM.

Brain fog and sleep disturbances have been on the spotlight. In patients with recent-onset VM, worse vestibular symptoms were linked to poorer learning [11]. Cognitive scores were worse in 61 VM compared with 30 healthy participants (HP) and may be multifactorial, arising from the interplay between vestibular impairment, mood disturbances, and demographic factors such as age and education [12]. A case-control study of 50 VM and 50 HP found that VM is associated with worse results interictally in multiple domains, which correlated with symptom burden [13]. In a prospective study, dizziness handicap correlated with worse sleep quality [14], and the expression of genes associated with the circadian rhythm may be lower in VM [15].

Longitudinal progression of VM and chronobiology may be important time-related variables to understand the VM spectrum. A 3-year prospective study found no changes in vertigo duration in 32 VM [16]. Scales can be useful for tracking symptom change and clinical impact in longitudinal follow-up [17]. However, they are less suited to understand the underlying neural or vestibular mechanisms that drive those symptoms. Biological rhythms such as the menstrual cycle may modulate susceptibility to VM, with several studies exploring hormonal patterns. Perimenstrual VM attacks [16] have been described, and menopause was a risk factor for ongoing attacks [6^▪^]. The potential pathophysiological links between VM and mood disorders in a cohort with very high prevalence of anxiety [18] have also been interpreted with caution, as the predominance of middle-aged females may imply perimenopausal fluctuations as trigger [19], irrespective of the direction of causality. Spatial anxiety is correlated with orientation abilities in HP and VM, although sense of direction was more affected in VM than those with other vestibular disorders [20], and alteration of spatial perception in subjective visual vertical was found interictally in 31 VM [21].

Ambulatory eye-movement recording is a promising way to characterize ictal and interictal features [22]. VM patients exhibit more spontaneous nystagmus than other headache types [23]. Reports listing VM as a common cause of downbeat nystagmus [24], likely use downbeat nystagmus descriptively, as nystagmus with a downward fast phase, rather than diagnostically, a phenomenological approach that is clinically useful but not aligned with standardized definitions [25]. Eye movements may be indeed a key element in VM pathophysiology: specific motion illusions mirror ocular drifts or nystagmus on video-oculography, including in nitroglycerin-provoked episodes [26], although the observations of this oculo-perceptual congruence are still preliminary and need systematic characterization.

## DIFFERENTIAL DIAGNOSIS AND COMORBIDITIES

Comorbidities and other vestibular disorders make the differential diagnosis of VM, a great mimicker, challenging. VM attacks can mimic other conditions such as benign positional paroxysmal vertigo (BPPV) or Meniere's disease (MD) [27]. Persistent postural perceptual dizziness (PPPD) is one of the main differential diagnoses in the context of daily episodes. These two entities entail a complex differentiation and overlap, since up to 60% of PPPD have co-existing VM [28] and the majority of VM can present almost daily symptoms [6^▪^,^29^]. A quarter of PPPD patients disclosed VM as a precipitating factor [30], and a migrainous background could predispose to symptom chronification. Assuming that the challenging diagnostic boundaries have been applied appropriately, mechanistic similarities are being identified between VM and PPPD. PPPD patients, like those with VM, exhibit impaired habituation in auditory middle latency responses, which would share underlying sensory processing abnormalities, consistent with the high prevalence of migraine in PPPD and the overlap in functional network alterations [31]. Motion sickness, classically associated with migraineurs, could be useful to differentiate VM in the vestibular setting [29,32,33].

Up to 22% of people with MD have migraine [34]. MD may present more rotational vertiginous symptoms compared to VM [5]. Motion sickness again emerges as a useful symptom in the differential diagnosis, with a much higher prevalence in VM vs. MD patients [35]. Overlapping features, such as tinnitus, are present in up to 75% of VM patients [36]. Migraine associated with sensorineural hearing loss, aural fullness or peri/intra auricular headache could be encountered in otorhinolaryngology clinics [37] rather than in headache or vestibular specialist clinics. Vestibular-auditory symptoms have a significant overlap in general migraine patients [38], which may determine different VM phenotypes not usually seen by the general or headache neurologist [39^▪^].

Migraine biology also plays a role in positional nystagmus and BPPV-like syndromes. Positional nystagmus patterns classically attributed to BPPV have in fact been shown to correspond to VM [40^▪^]. A prospective study found a higher probability of BPPV recurrence in migraineurs [41], who could experience more disabling BPPV symptoms [42].

Although vestibular neuritis is not generally regarded as showing a strong sex difference in incidence, a recent study found a higher prescription of corticosteroids in women [43]. This subtle asymmetry invites consideration of whether part of what we label as neuritis might be misclassified vestibular migraine or another transient vestibular disturbance.

Infections, such as COVID-19, may play an important role in the onset of vestibular symptoms. In a study of post-COVID headache, dizziness was reported in a third of patients; more frequently than cognitive complaints [44]. COVID-19 appears capable of worsening both dizziness and migraine in VM, particularly among women and those with preexisting autoimmune tendencies [45]. This link may not be coincidental: type 1 immune lymphoid responses seem to differ between migraine and VM [46], and the immunological profile of VM diverges from that of Meniere's disease MD [47]. Autoimmune comorbidities seem to be, indeed, more prevalent in participants with VM vs. CM [48], and both autoimmune conditions and Postural Orthostatic Tachycardia syndrome (POTS) independently predicted a VM phenotype [49]. Up to 84% of POTS patients have migraine, more frequent and disabling vestibular symptoms [50]. Taken together, these observations evoke a broader neuro-immuno-autonomic continuum, of which VM may be one of the most intricate expressions.

## NEUROIMAGING

Neuroimaging studies of vestibular migraine offer a fascinating, if still fragmented, window into the brain's attempt to reconcile motion, space, and perception. In structural neuroimaging, a recent coordinate-based meta-analysis of five voxel-based morphometry studies (103 VM, 107 HP) revealed grey matter volume reductions centered on the left rolandic operculum and posterior insula [51], regions integral to vestibular and interoceptive integration. Another study reported that grey matter morphological networks in VM appear more locally clustered yet less efficiently connected, particularly within the left superior temporal gyrus. A wider set of regions also showed reduced structural coupling. Although these alterations did not translate into direct clinical correlations, machine learning algorithms could differentiate VM from healthy participants in most cases, suggesting that the neural changes of VM may be subtle but distinctive [52]. Spectroscopy analysis in a small study on 15 VM participants showed a left posterior thalamus choline value lower than those with nonvestibular migraine [53], adding a metabolic dimension to these structural findings.

A meta-analysis of nine functional neuroimaging studies including 251 VM and 257 healthy participants identified a distributed pattern of altered brain activity. Compared with controls, VM showed reduced activation in temporal and cingulate cortices, regions linked to self-motion and interoception, and increased activity in parietal and frontal association areas. The degree of dizziness-related disability correlated with increased activity in the precuneus, while higher headache and vestibular burden were associated with reduced midcingulate/paracingulate activity, implicating networks involved in spatial orientation, sensory integration, and cognitive control [54].

Recent functional imaging studies further suggest that VM is less a disorder of isolated regions than of altered communication between them. Using a combination of resting-state arterial spin labelling (ASL) and fMRI, a study demonstrated increased baseline cerebral blood flow in the sensorimotor cortices, frontal association areas, and bilateral insulae, key regions in sensory integration and interoceptive awareness. Connectivity analyses revealed stronger coupling between primary sensorimotor regions and visual processing areas, yet reduced connectivity between the left insula and subcortical/limbic structures, alongside an enhanced synchrony between the left insula and right fusiform gyrus, involved in complex visual recognition. These shifts in network balance correlated with the frequency of migraine and vestibular symptoms, as well as with disease duration [55], suggesting that the neural architecture of VM adapts (or perhaps maladapts) over time.

The temporal dynamics of brain activity were compared in 57 individuals with VM and 88 healthy participants. Spontaneous BOLD fluctuations in VM were unusually labile, showing time-varying activity and concordance within the sensorimotor cortex, but less stable local synchrony in right temporal pole, posterior cerebellum, angular gyrus, and middle occipital gyrus [56]. Such findings point to a network not over or underactive, but dynamically unstable.

At a more peripheral level, inner ear MRI studies have sought to explore the structural differences between VM and MD. The visibility of endolymphatic sac and duct in 67 VM participants following the administration of intravenous gadolinium, which was impaired in participants with MD, and inversely correlated with the volume of endolymphatic hydrops. These structures were normally visible in the VM and HP group [57]. The pattern of hydrops also differed between conditions, with a higher distribution in the vestibulum in VM in comparison with the predominant distribution in the cochlea in MD [58], supporting the notion that, despite clinical overlap, the underlying pathophysiology diverges at the level of labyrinthine homeostasis. Further corroborating the distinction, endolymphatic hydrops occurred in 5% of VM participants compared to 92% of those with MD in a contrast-enhanced 3T FLAIR MRI using intratympanic gadolinium [35]. A smaller prospective series reached similar conclusions: none of the 16 VM participants developed cochlear or vestibular endolymphatic hydrops, nor asymmetrical perilymphatic enhancement [59]. Together, these imaging studies suggest that while vestibular migraine may mimic Meniere's disease MD symptomatically, its structural footprint within the inner ear is either absent or fundamentally different.

## TREATMENTS

Differences in treatment amongst sub-specialties remains an area of discussion. Relying solely on the management of potential triggers risks leaving VM patients under-treated, particularly in nonheadache care, where more than half of patients ultimately require preventive therapy despite sensible initial nonpharmacological management [60].

Study designs vary across specialties and endpoints are often vestibular-nonspecific, limiting comparability. Studies led by headache specialists may align with contemporary methodological guidance in the field [61], whereas trials developed in other specialties may reasonably prioritize different frameworks. Results are, therefore, not always directly comparable or generalizable.

A randomized-controlled trial of duloxetine (*n* = 33), which is not a standard migraine treatment, reported short-term reduction in attack frequency vs. placebo. Attack frequency assessed over just one week and unknown doses [62], make efficacy difficult to interpret. A small self-selected comparison of nortriptyline (10–40 mg) vs. lifestyle modifications and nutraceuticals, including unspecified doses of magnesium and riboflavin for 4 weeks yielded significant reductions in dizziness and stress in the lifestyle arm, and improved quality of life in both groups. While this study is a welcome attempt to compare pharmacological and lifestyle strategies, its findings are difficult to interpret. Older migraine preventives typically requires gradual titration and ≥8–12 weeks to judge efficacy. Moreover, the comparator is an active, multicomponent intervention with unspecified doses, so it is not a neutral control, and dose-response comparisons are impossible. Self-selection into arms, small sample size, and lack of randomization and blinding further constrain causal inference [63].

Monoclonal antibodies targeting calcitonin gene-related peptide (CGRP) now have direct evidence in VM, although more randomized-controlled trials (RCTs) are still needed [17]. In the first RCT for VM, galcanezumab significantly reduced Vestibular Migraine Patient Assessment Tool and Handicap Inventory (VM-PATHI) and DHI scores and lowered dizzy days compared to placebo [64]. Indeed, CGRP levels in VM are not different from other types of migraine [65^▪^]. A retrospective study in Japan assessed 12 patients on erenumab or galcanezumab vs. conventional Japanese treatments, alongside vestibular rehabilitation for 6 months. There was a significant improvement in dizziness handicap and frequency. Baseline autonomic dysfunction, assessed with head-up tilt test, was independently associated with greater response [*β* = 3.63; 95% confidence interval (CI) 0.21–7.06] [66]. The independent link between POTS and VM [48] and the positive association of autonomic dysfunction with treatment response suggests that stratifying vestibular migraine by autonomic phenotype when considering preventive options could be useful.

Onabotulinumtoxin-A (OBT-A) by PREEMPT protocol could reduce VM symptoms, as measured by the visual analogue scale, MIDAS and DHI from the first month in small a double-blinded RCT, without detectable changes in video-Head Impulse Test or cervical-vestibular evoked myogenic potentials [67^▪^]. Reports of improvement after right PFO closure or pulmonary arteriovenous malformation embolization are intriguing, but remain nonrandomized and short term [68].

Regarding symptomatic treatments, a study of rizatriptan 10 mg against placebo in 134 VM participants that treated 240 attacks did not significantly reduce vertigo or unsteadiness/dizziness at 1 h, although at 24 h it showed efficacy in unsteadiness/dizziness and motion sensitivity [69^▪^]. Acute efficacy in VM may unfold more slowly than in nonvestibular migraine.

## CONCLUSION

The latest research in VM underscores the enduring value of careful clinical anamnesis and detailed symptom phenotyping, alongside imaging and physiology as adjuncts. Current studies, heterogeneous though they are, converge on disturbed integration across vestibular, visual and interoceptive networks rather than focal damage. Importantly, VM shares many migraine-associated symptoms, including cutaneous allodynia and sensory hypersensitivities, reinforcing its place within the migraine spectrum. Emerging treatments (CGRP antibodies, onabotulinumtoxin-A) are promising, while acute responses may be slower and less robust than in typical migraine. Harmonized endpoints, vestibular-specific outcomes, and phenotype-guided trials, including autonomic and sensory-hypersensitivity profiles, should bridge research and practice and, crucially, improve the day-to-day life of patients.

## Acknowledgements

*None.*

### Financial support and sponsorship

*None.*

### Conflicts of interest

*P.J.G. reports, over the last 36 months, personal fees for consulting from Aeon Biopharma, Abbvie, CoolTech LLC, Dr Reddy's, Eli-Lilly and Company, Epalex, Ipsen, Kallyope, Linpharma, Lundbeck, Orion Pharma, Pfizer, PureTech Health LLC, Satsuma, Seaport Pharma, Shiratronics, and Teva Pharmaceuticals.*

*M.D.V.M. does not have any conflict of interest relevant to this manuscript.*

## REFERENCES AND RECOMMENDED READING

Papers of particular interest, published within the annual period of review, have been highlighted as:

▪ of special interest
▪▪ of outstanding interest
